# The Sexual Disabilities of Man and Their Treatment

**Published:** 1911-01

**Authors:** 


**The Sexual Disabilities of Man and Their Treatment.** By **Arthur Cooper,** Consulting Surgeon to the Westminster Dispensary. London: Second edition. 12 mo, pp. 204. Paul B. Hoeber, publisher, 69 East 59th Street, New York. (Cloth, $2.00.)

The present volume is based mainly on what has been observed in the practice of the author during the last thirty years, and it is hardly necessary to say that it does not pretend to be exhaustive. Physicians, however, will find it a useful guide in dealing with a class of patients whose ills are very real, and often difficult to cure. It will also be of use to the student with little knowledge of the subject, one which receives but scanty recognition in the medical schools of this country. J. A. R.

